# Different media and supplements modulate the clonogenic and expansion properties of rabbit bone marrow mesenchymal stem cells

**DOI:** 10.1186/1756-0500-1-53

**Published:** 2008-07-28

**Authors:** Simone Lapi, Francesca Nocchi, Roberta Lamanna, Simona Passeri, Mariacarla Iorio, Aldo Paolicchi, Patrizia Urciuoli, Alessandra Coli, Francesca Abramo, Vincenzo Miragliotta, Elisabetta Giannessi, Maria Rita Stornelli, Renato Vanacore, Giulia Stampacchia, Guido Pisani, Luciano Borghetti, Fabrizio Scatena

**Affiliations:** 1 Cell Biology and Tissue Regeneration Laboratory-Immunohematology 2 Unit – Azienda Ospedaliera Universitaria Pisana, Pisa, Italy; 2 Department of Veterinary Anatomy, Biochemistry and Physiology, University of Pisa, Italy; 3 Department of Experimental Pathology-University of Pisa, Italy; 4 Department of Animal Pathology, University of Pisa, Italy; 5 Private practitioner – Centro veterinario 19033 Molicciara – Italy; 6 Department of Neuroscience – Azienda Ospedaliera Universitaria Pisana, Pisa, Italy

## Abstract

**Background -:**

Rabbits provide an excellent model for many animal and human diseases, such as cardiovascular diseases, for the development of new vaccines in wound healing management and in the field of tissue engineering of tendon, cartilage, bone and skin.

The study presented herein aims to investigate the biological properties of bone marrow rabbit MSCs cultured in different conditions, in order to provide a basis for their clinical applications in veterinary medicine.

**Findings -:**

MSCs were isolated from 5 New Zealand rabbits. Fold increase, CFU number, doubling time, differentiation ability and immunophenotype were analyzed.

With the plating density of 10 cells/cm^2 ^the fold increase was significantly lower with DMEM-20%FCS and MSCs growth was significantly higher with αMEM-hEGF. The highest clonogenic ability was found at 100 cell/cm^2 ^with MSCBM and at 10 cell/cm^2 ^with M199. Both at 10 and 100 cells/cm^2^, in αMEM medium, the highest CFU increase was obtained by adding bFGF. Supplementing culture media with 10%FCS-10%HS determined a significant increase of CFU.

**Conclusion -:**

Our data suggest that different progenitor cells with differential sensitivity to media, sera and growth factors exist and the choice of culture conditions has to be carefully considered for MSC management.

## Background

Bone marrow contains at least two major stem cell lineages, hematopoietic [1,2] and mesenchymal (stromal) cells [3-5]. A standard in vitro assay for bone marrow stromal cell activity is the Fibroblastic Colony-Forming Unit (CFU-F) assay in which adherent fibroblastic cells are cultured by plating bone marrow cells either directly or following gradient separation [6]. The phenotype of these cells appears to vary depending on the culture conditions and the specific cell preparation [7,8]. Typically a broad range of colony sizes has been obtained, with varying growth rates and different cell morphologies [9], probably reflecting the presence of a mixed population of multi-, bi-, and unipotential progenitors [10,11], whose features and biological properties have been only partially investigated.

The aim of the present study was to compare the basic biological properties of the cells obtained with different culture media, supplements, and protocols of rabbit Mesenchymal Stem Cell (rMSC) culture, in order to establish the optimal culturing conditions thus providing a basis for further studies on the biological heterogeneity of rMSC.

## Methods

### Isolation, expansion and differentiation of rMSC

Five New Zealand female rabbits were included in the study under guidelines determined by the Local Ethical Committee.

Five mL samples of bone marrow aspirates from femur were drawn and treated to induce haemolysis. The remaining nucleated cell suspensions were centrifuged at 500 g for 10 minutes and the pellets were resuspended in low glucose DMEM supplemented with 10% fetal calf serum (FCS), 10 U/mL penicillin G, 10 μg/mL streptomycin, 2 mM L-glutamine.

Cells were counted and resuspended in standard culture medium (SCM): α-MEM, 20% FCS, 100 U/mL penicillin, 100 μg/mL streptomycin, 2 mM L-glutamine. Plating concentration was 10^5^cells/cm^2 ^in 25 cm^2 ^tissue culture flasks. After 24 hours the non adherent cells were removed by washing with PBS. Fresh SCM was added twice a week up to 90% confluence (passage 0, P0); at P0, CFU-F were counted by visual examination to evaluate the rMSC number in the primary culture. Cells were then harvested for further expansion and re-plated at 5000 cells/cm^2^. At the end of each passage (90% confluence) cells were counted. Cell Doubling (CD), Cumulative Population Doublings (CPD) and Doubling Time (DT) values were calculated following established formulae.

Osteogenic, chondrogenic and adipogenic differentiation were performed following standard protocols.

### Determination of influence of culture passage and plating density on cell proliferation and clonogenic potential

Aliquots of rMSC from different culture passages (P1, P2 and P3) were assayed for cell proliferation (fold increase), and clonogenic ability (CFU-F assay).

Cells were plated at 10 cells/cm^2^, 100 cells/cm^2 ^and 1000 cells/cm^2 ^in SCM in 12-well tissue culture plates in duplicate. Every day for a week, cells from each culture density (in triplicate) were detached and counted in a haemocytometer. Viability was assessed by 0.4% Trypan Blue exclusion test. Fold increase was calculated dividing the number of harvested cells at 90% confluence by the number of plated cells.

To evaluate the clonogenic potential the CFU-F assay was performed as follows: rMSC were seeded in SCM, at 10 cells/cm^2^, 100 cells/cm^2 ^and 1000 cells/cm^2 ^in 6-well tissue culture plates. Colonies were counted on day 7 and 10. Cells were then stained with Crystal Violet (0.5%) in methanol at RT for 10 minutes, washed twice with PBS and visually counted.

### Selection of different culture conditions and media for rMSC expansion and clonogenic ability

P3 cells were plated at 10 cells/cm^2 ^and 100 cells/cm^2 ^either in α-MEM, or DMEM, or Medium199 (M199), or Mesenchymal Stem Cells Basal Medium (MSCBM). FCS 20%, Penicillin 100 U/mL, Streptomycin 0.1 mg/mL and L-Glutamine 2 mM were added to α-MEM, DMEM and M199. To evaluate the effects of growth factor supplementation, 10 ng/mL human Epidermal Growth Factor (hEGF) or 10 ng/mL fibroblast growth factor (bFGF) or insulin 5 ug/mL were added to all media, with the exception of MSCBM.

To assess the effects of different serum supplements, α-MEM, DMEM and M199 containing Penicillin 100 U/mL, Streptomycin 0.1 mg/mL and L-Glutamine 2 mM, were supplemented either with 20% FCS or 10% FCS – 10% Horse Serum (HS).

After expansion for 8 days cell were counted. Fold increase and CFU-F number were calculated.

### Epitope analysis of rMSC: FACS and immunochemical analyses

FACS analyses were performed on P3 cells by using anti-MHC I, anti-MHC II, anti-CD14, anti-CD45, anti-CD44, anti-β-1-integrin and anti-CD90 mouse monoclonal antibodies. As secondary antibody a FITC goat anti mouse IgG was used. P3 cells were used for immunohistochemistry. As primary antibodies anti-β-1-integrin, anti-CD90, anti-MHC I, anti-MHC II, anti-vimentin, anti-α-smooth-actin, anti-cytokeratin, anti-desmin were used. Reactivity was visualized by streptavidine-peroxidase method.

## Results

### Isolation, expansion and differentiation of rMSC

The average number of marrow cells collected was 2.6 ± 1.1 × 10^7 ^(n = 5). The CFU number calculated at P0 was 170 ± 72 (n = 5) and there was a positive correlation between the number of collected cells and the amount of obtained CFUs at P0 (r = 0.89 p < 0.01).

**Figure 1:**
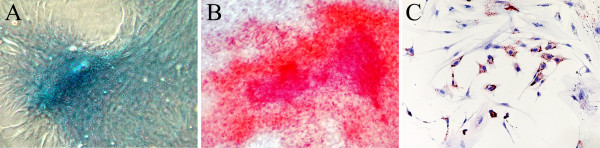
rMSC differentiated into chondrocyte, osteocyte, and adipocyte cells. Chondrogenic differentiation detected by Alcian blue staining (A), osteogenic differentiation detected by Alizarin Red staining (B), adipogenic differentiation detected by Oil Red O staining (C) at light microscopy.

The average of rMSC from each explant at the end of P0 was 11.2 ± 4.9 × 10^5 ^(n = 5), corresponding to 12.7 population doublings. The CPD (22.1 ± 1.69) positively correlated with the number of CFU at P0 (r = 0.76 p < 0.01). Chondrogenic, adipogenic and osteogenic differentiation was achieved (Fig. 1).

### Determination of influence of culture passage and plating density on cell proliferation (fold increase) and clonogenic potential (CFU-F assay) (Fig. 2)

**Figure 2:**
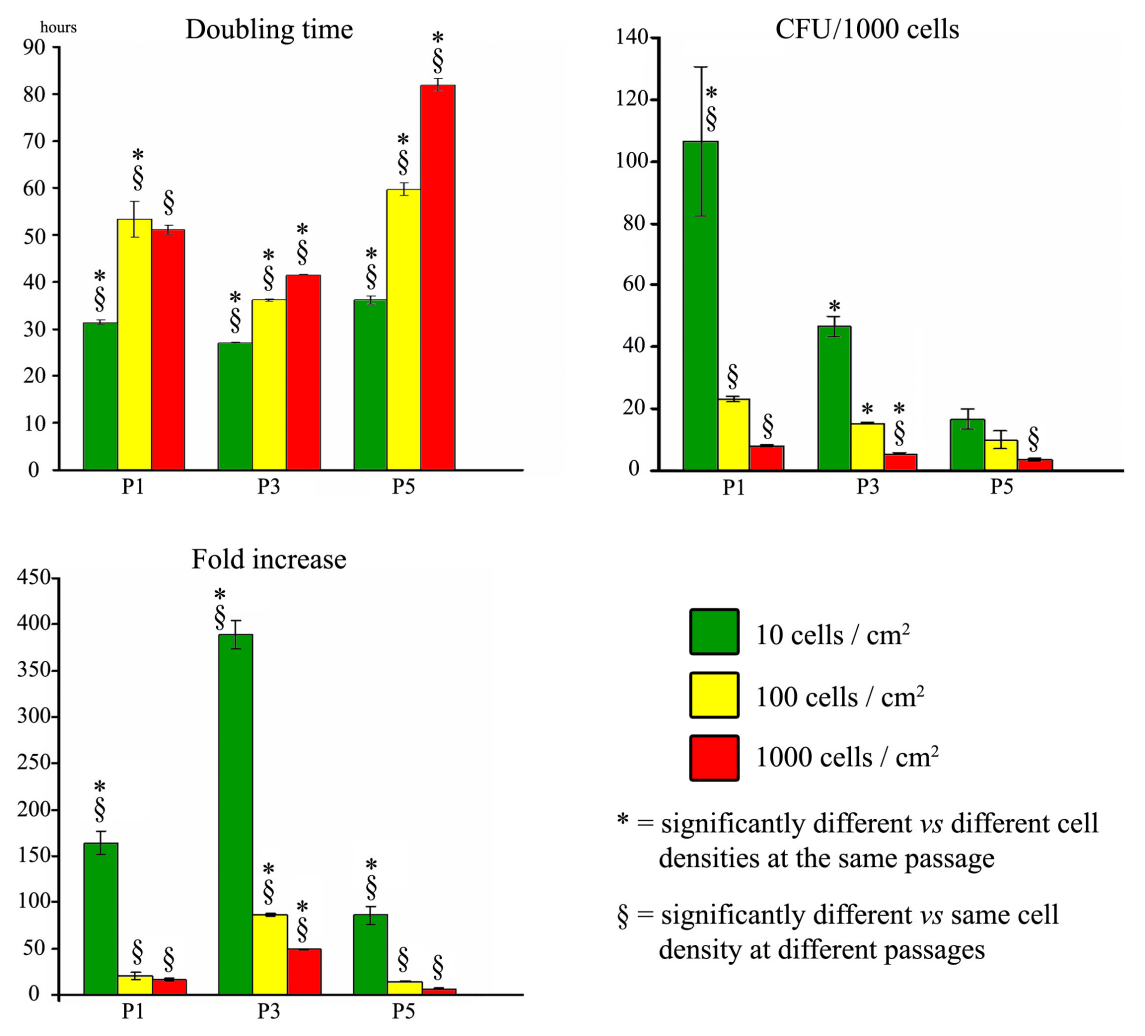
Effect of plating density and culture passage on rMSC doubling time, clonogenic ability and expansion potential.

Doubling time at P3 was always significantly lower than at P1 and P5 (p < 0.001), and within each passage it was significantly affected by cell density.

Fold increase value was in inverse relation to the number of cells plated at each culture passage at all cell densities (p < 0.001).

The number of CFU/1000 cells was in inverse relation to the number of cells plated; in particular at P3 a significant difference was observed at all cell densities (p < 0.01 10 cells/cm^2 ^and 1000 cells/cm^2^, p < 0.05 100 cells/cm^2^), while at P1 a significant difference was observed only at a plating density of 10 cells/cm^2^. The number of CFU at P1 was significantly higher in comparison with the other passages at 1000 cells/cm^2 ^(p < 0.001 vs. P5 and p < 0.01 vs. P3), at 100 cells/cm^2^(p < 0.01 vs. P5, and p < 0.05 vs. P3) and at 10 cells/cm^2 ^(p < 0.01 vs. P5, and p < 0.05 vs. P3).

### Effects of different culture conditions and media on rMSC expansion (Fig. 3A, B) and clonogenic ability (Fig. 3C, D)

**Figure 3:**
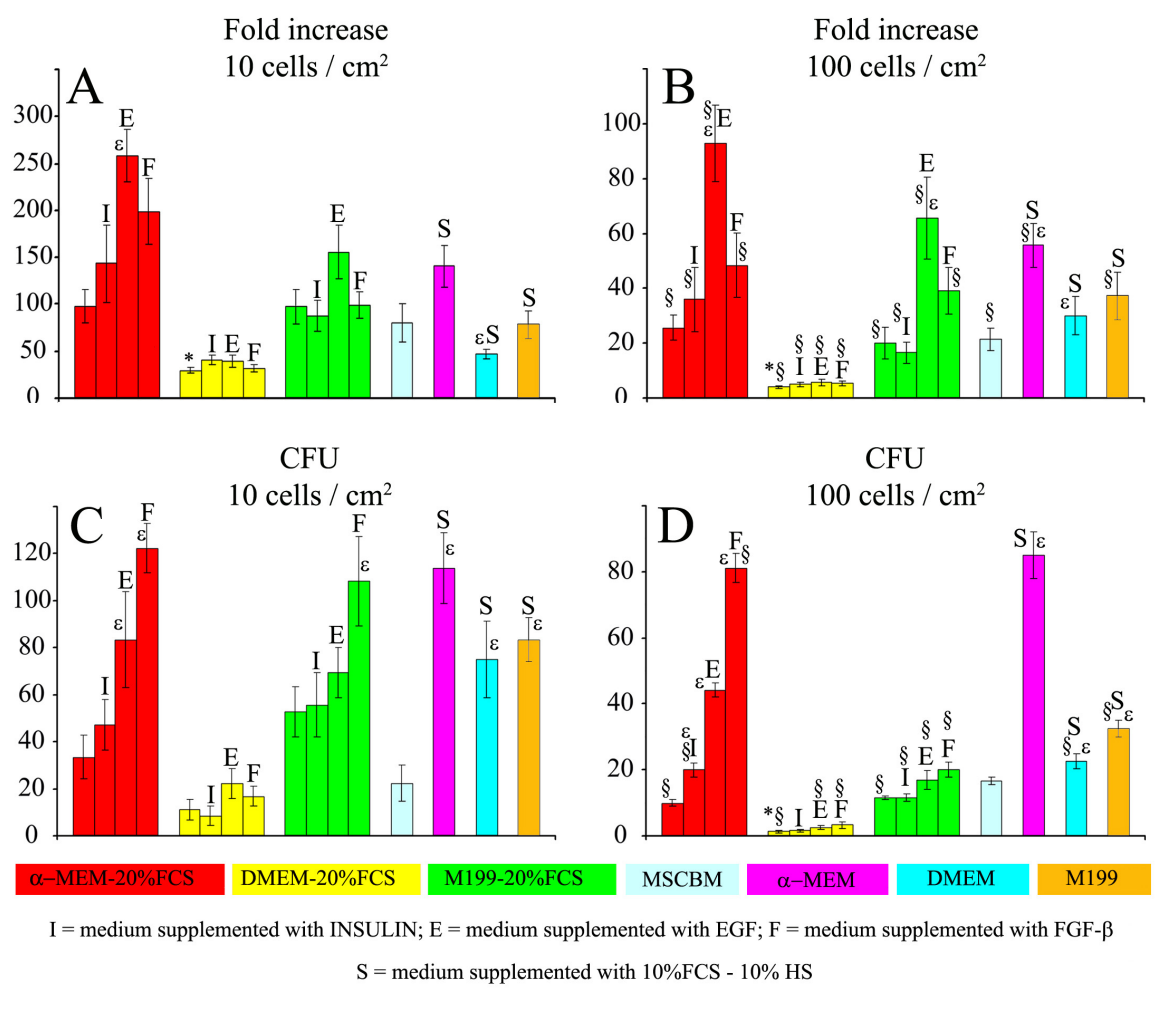
Evaluation of the effects of culture media, serum and growth factors on rMSC. A: fold increase at 10 cells/cm^2^; B: fold increase at 100 cells/cm^2^; C: CFU number at 10 cells/cm^2^; D: CFU number at 100 cells/cm^2^. * = significantly different from the other basal media w/o supplements; **ε **= significantly different from the corresponding basal medium w/o supplements; **§ **= significantly different from the corresponding medium at 10 cells/cm^2 ^plating density.

With plating density of 10 cells/cm^2 ^total fold increase ranged from 80 to 100 in all media except than DMEM-20%FCS which showed a 30-fold increase of cell number, significantly lower (p < 0.05) than all other media. With a plating density of 100 cells/cm^2 ^the fold increase was approximately 4–5 times lower in all media (p < 0.05 vs. respective controls), and the fold increase of DMEM was significantly lower than all other media (p < 0.01 *vs *α-MEM, p < 0.05 *vs *MSCBM and M199).

In α-MEM and M199 and not in DMEM, proliferation was significantly enhanced by EGF but not by insulin and FGF. The effect was partially dependent on the plating density, as it was observed in α-MEM and M199 at 100 cells/cm^2 ^(p < 0.01 *vs *control). At 10 cells/cm^2 ^a significant increase (p < 0.01) was found in α-MEM. The cell growth of rMSC in α-MEM supplemented with EGF was significantly higher than in MSCBM (p < 0.005) at 10 cell/cm^2^.

The substitution of 20%FCS with 10%FCS and 10%HS caused a significant increase of cell proliferation in α-MEM (p < 0.05) and DMEM (p < 0.05) at 100 cells/cm^2^. At 10 cells/cm^2 ^a significant decrease of fold increase was found for DMEM (p < 0.05).

Culture media influenced the number of colonies formed, the highest clonogenic ability being found at 100 cell/cm^2 ^with MSCBM (p < 0.001) and at 10 cell/cm^2 ^with M199 (p < 0.05 vs. all other media). The lowest value was found in DMEM both with 10 and 100 cells/cm^2 ^(p < 0.05 and p < 0.001, respectively).

Both at 10 cells/cm^2 ^and 100 cells/cm^2 ^in α-MEM the highest increase of CFU was found with FGF, which showed to be superior to EGF (p < 0.001 at 100 cell/cm^2 ^and p < 0.05 at 10 cell/cm^2^) and insulin (p < 0.001 at 100 cells/cm^2 ^and p < 0.01 at 10 cells/cm^2^). M199 showed a significant increase of CFU (p < 0.05 in 100 cell/cm^2 ^e p < 0.05 in 10 cell/cm^2^) when supplemented with FGF.

The clonogenic ability of rMSC in α-MEM supplemented with FGF was significantly higher than in MSCBM (p < 0.0001 both at 100 cells/cm^2 ^and 10 cells/cm^2^).

All media, supplemented with 10%FCS – 10%HS produced a significant increase of CFU (p < 0.05).

### Epitope analysis of rMSC: FACS and immunochemical analyses

rMSC from all animals were positive for CD29, and CD44. They did not express monocyte-macrophage antigens such as CD14 and CD45. The adherent cells were negative for MHC I and MHC II. Immunohistochemical analysis confirmed the positivity for CD29 and the negativity for CD90, MHC I and MHC II. rMSC resulted positive for vimentin, α-smooth actin and desmin while they were negative for cytokeratin, as showed in table 1 in comparison with data already reported for humans and rodents.

**Table 1:** Cell surface epitopes on rMSC and other rodents (rat, mouse)/human MSC

**Cell surface epitope**	**Rabbit MSC**	**Rodent/human MSC**
CD14	-	-
CD29	+	+
CD44	+	+
CD45	-	-
CD90	-	+/-*
MHC I	-	+/-**
MHC II	-	-
Vimentin	+	+
Desmin	+	+
α-smooth-actin	+	+
Cytokeratin	-	-

* negative in mouse

** MHC I^+/- ^phenotype was found in human umbilical cord perivascular cells

## Discussion

We found that at both 10 and 100 cells/cm^2 ^plating density was significantly lower with DMEM than α-MEM. In all basal media the decrease of cell density improved fold increase; moreover with the exception of MSCBM this improvement was followed by a proportional increase of clonogenic ability. The average number of cells formed by each clone at both plating densities remained substantially the same. In MSCBM we observed a significant improvement of fold increase at 10 cells/cm^2 ^while no substantial variation was found on the clonogenic ability. Therefore in this case the highest cell number seems to depend on a greater proliferation rate of the clonogenic units.

In terms of clonogenic ability, 10%FCS – 10%HS supplementing performed significantly better than 20%FCS when cells were grown in α-MEM, M199 or DMEM at both plating densities; the same performance enhancement was not always detectable for cell proliferation.

EGF was able to enhance the fold increase, the highest performance being found with α-MEM+EGF at 10 cells/cm^2^. This latter information suggests that cell density strongly influences the culture requirements of rMSC and low-cell density plating minimizes the requirement of supplements.

We also found that the expansibility of rMSC was predictable on the basis of a CFU-F assay, likely reflecting the number of rMSC in the bone marrow sample, as already suggested [12,13]. The strong positive correlation found in our study between CFU-F number at P0 and population doublings confirms that CFU-F assay can be useful for predicting the cell suitability for extensive expansion.

As reported for humans [5], rats [7] and mice [14] we found that rMSC expansion increased dramatically when cells were plated at low density: this might be due to an higher cell-to-cell contact inhibition or factors synthesized as a consequence of higher cell concentration able to decrease the proliferative ability of the population [15].

Moreover, in the first passages, we found that the clonogenic ability of rMSC was in inverse relation to the plating density. The doubling time decreased from P1 to P3 and it could depend on the progressive increase of rapidly dividing rMSC which are thought to represent a minute fraction of the cells plated in the first passages [16].

We observed at least two morphologically distinct cells: spindle shaped and broad cells, corresponding to the previously described type I and type II cells [17,18].

It has been reported that CD90 and MHC I are generally expressed in mesenchymal stromal cells [19], and CD90 expression shows a great variability [14]. Recently it has been reported that mesenchymal progenitors in the umbilical cord do not express MHC I under particular conditions [20]. In our study CD90 and MHC I were both negative. The variability of the reported surface markers on mesenchymal cells may depend on different stage of commitment, or just on the ex-vivo culturing conditions.

The immunoreactivity obtained against CD44, CD29, desmin, vimentin and α-smooth actin, as well as the negative reaction for CD14, CD45, MHC II, cytokeratin concord with data reported for humans and rodents.

Taken together, our data suggest that different progenitor cells with differential sensitivity to media, sera and growth factors exist and may be expanded in selected conditions. The choice of culture media, supplements and serum compositions has to be carefully considered to expand rMSC since it can lead to considerable variation in yield and CFU number.

## Authors' contributions

PU, FN, RL and SP carried out the MSC isolation and differentiation. GS, MI and RV designed and performed the FACS analyses. AC, FA, VM, EG and MRS designed and performed the immunochemical analyses. GP and LB provided rabbit bone marrow samples. AC, VM, FA, SP, SL and AP contributed to write and revise the manuscript. SL, AP, AC, FA and FS conceived the entire experimental design and provided funding.
